# Mother-infant bonding and postpartum depression during the COVID-19 pandemic — a risk for nurturing care and child development

**DOI:** 10.1590/1984-0462/2024/42/2022151

**Published:** 2023-07-10

**Authors:** Bárbara Portela Diniz, Sandra Josefina Ferraz Ellero Grisi, Danton Matheus de Souza, Ana Paula Scoleze Ferrer

**Affiliations:** a Universidade de São Paulo, São Paulo, SP, Brazil.

**Keywords:** Pandemics, Bonding, Child development, Postpartum depression, Pandemias, Vínculo, Desenvolvimento infantil, Depressão puerperal

## Abstract

**Objective:**

The COVID-19 pandemic Increased the risk of impairing the mother-infant bonding. The objectives of this study were to evaluate the early bond established between mother and infant and postpartum depression (PPD) in pregnancies that occurred during the pandemic period, to identify the factors that may have influenced these outcomes and to verify if there was an association between bonding and probable PPD.

**Methods:**

This is a cross-sectional study of postpartum women from a public maternity hospital in the city of São Paulo conducted from February to June 2021, involving 127 mother-baby dyads. The initial data were collected in the immediate postpartum period and between 21–45 days after birth, using a semi-structured questionnaire on sociodemographic characteristics, gestational and birth conditions, and baby characteristics; the Edinburgh Postnatal Depression Scale (EPDS) and Postpartum Bonding Questionnaire (PBQ) were used to evaluate PPD and bonding, respectively.

**Results:**

The presence of probable PPD and unplanned pregnancies were associated with higher PBQ score and risk to impaired bonding (p = 0.001 and p = 0.004, respectively). EPDS showed a high prevalence of PPD (29.1%) and was not associated with any Studied variable. Probably, this high prevalence of probable PPD was due to the context of insecurity secondary to the pandemic.

**Conclusions:**

We observed an increase in the prevalence of probable PPD and unplanned pregnancies during the first 18 months of the pandemic, which were associated with worse scores in mother-infant bonding. The impaired bond can affect the future development of children born during this period.

## RESUMO

**Objetivo:**

A pandemia do COVID-19 aumentou o risco de o vínculo entre mãe-bebê ser prejudicado. Os objetivos deste estudo foram avaliar o vínculo mãe-bebê estabelecido precocemente e a depressão puerperal (DP) em gestações que ocorreram durante a pandemia, identificar os fatores que podem ter influenciado esses resultados e verificar se houve associação entre o vínculo e a provável depressão puerperal.

**Métodos:**

Foi realizado um estudo transversal com puérperas de uma maternidade pública da cidade de São Paulo, no período de fevereiro a junho de 2021, envolvendo 127 díades mãe-bebê. Os dados foram obtidos no puerpério imediato e 21 a 45 dias após o parto, utilizando-se um questionário semiestruturado sobre as características sociodemográficas, as condições de gestação e nascimento e características do recém-nascido; a escala de depressão puerperal de Edimburgo (EPDS) e o questionário de vínculo pós-parto (PBQ) foram utilizados para avaliar a presença de provável DP e o vínculo mãe-bebê, respectivamente.

**Resultados:**

A presença de provável DP e as gestações não planejadas foram associadas a maiores escores no PBQ e no risco de vínculo prejudicado (p = 0,001 e p = 0,004, respectivamente). A escala de Edimburgo mostrou alta prevalência de risco de depressão puerperal (29,1%) e não foi associada a nenhuma variável estudada. Provavelmente, a alta prevalência de provável DP foi secundária ao contexto de insegurança decorrente da pandemia.

**Conclusões:**

Observamos aumento na prevalência da provável DP e das gestações não planejadas durante os primeiros 18 meses da pandemia, os quais estiveram associados a piores escores no vínculo mãe-bebê. O prejuízo no vínculo pode afetar o desenvolvimento futuro das crianças geradas durante esse período.

## INTRODUCTION

Mother-infant bonding is defined as the reciprocal relationship established between mother and child through psychological and physical interaction, permeated by affection, security, empathy, and emotional motivation.^1,2^ It is considered the most important psychological process after birth, the basis for secure attachment, and essential to ensure nurturing care and adequate neurophysiological, physical, and psycho-emotional development. The better the bond, the more sensitive and responsive the caregiver is to the child’s needs.^1,3,4^ The mother-infant relationship begins during pregnancy, but intensifies soon after birth due to physical, hormonal, and emotional transformations related to the pregnancy-puerperal cycle. Some studies show that, in the first days after birth, mothers can develop a way to identify the needs of their own children through physical and visual contact, which indicates that the mother-infant bond is being successfully established.^5^

Countless factors may influence the establishment, maintenance, and quality of the bond:

1. Those related to the mother’s physical and mental health, particularly the presence of postpartum depression (PPD);
2. Those related to the children, such as their temperament and the presence of any conditions associated with greater fragility, such as prematurity; and
3. Socioenvironmental factors, such as socioeconomic conditions, the presence or absence of social support, and the quality of the marital relationship.^6^

The COVID-19 pandemic has triggered several social, economic, and psychological changes affecting the emotional well-being of families, entailing several consequences, and establishing an unfavorable context for the exercise of parenthood. Several studies have reported increased parenting stress, anxiety, concern, relational conflicts, fear, sleep disorders, and increased PPD, all of which have the potential to compromise the quality of the bond between parents and child.^7–9^ From these reports, the impacts of the pandemic on child development have become a focus of concern.

Many authors have reported an increase in PPD. Galletta et al.^9^ found high rates in Brazil (38.8%), as did Mariño-Narvaez et al.^10^ (37.3%) in Spain, corresponding to more than twice compared to pre-pandemic levels. Problems in mother-infant bonding have been less studied, but an increase in their prevalence has also been reported. Suzuki^11^ compared two samples of mothers before and during the pandemic and found 2.56 times higher chances of an altered score in the assessment of bonding one month after birth. In the same way, Fernandes et al.^12^ found worse scores on the Postpartum Bonding Questionnaire (PBQ) performed in the first year of life in Portuguese mothers, relating the worst scores to parental stress. These authors showed a worsening of the bond after the pandemic onset, and it is very important to understand which factors were related to these undesirable outcomes.

Understanding the effects of the pandemic on the mental health of caregivers and on children and identifying the main factors that can impair parenting are fundamental for future research on the consequences of this on child development, besides directing the readjustment of care practices that have been considered fundamental to mitigate future impacts.^13^

The objectives of this study were to evaluate the early bonding established between mother and infant and PPD in pregnancies that occurred during the pandemic period and to identify the factors that may have influenced these outcomes and to verify if there was an association between bonding and probable PPD.

## METHOD

The study was conducted with postpartum women at the University Hospital of the University of São Paulo (HU-USP). This is a public teaching hospital that serves the low-income population in São Paulo, the biggest city in Brazil, where about 200 deliveries per month of low-risk pregnancies usually occur.

**Figure 1.**
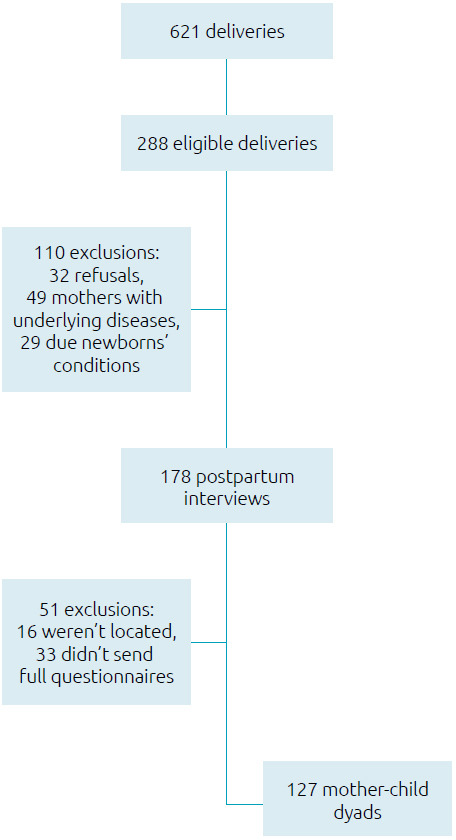
Consort diagram.

This was a cross-sectional study with a convenience sample composed of mothers who were on the first postpartum, carried out on days on which the main researcher attended the hospital: Mondays, Wednesdays and Fridays between February 7 and June 1, 2021 (therefore including pregnancies that occurred during the first 18 months of the pandemic), and who agreed to participate after signing the informed consent form. The exclusion criteria were conditions that could harm mother-infant bonding — mothers with underlying diseases or COVID-19 during childbirth, newborns with malformations, syndromes, twins or who had stayed in intensive care units (ICU). As there were changes in the organization of the health system due to the pandemic, there were fewer births during the study period (621 over four months) and a greater number of mothers with chronic diseases and high-risk pregnancies at the study hospital, who were excluded due to a greater risk of having PPD and/or bonding problems. Figure 1 summarizes the sample’s composition.

Data were collected from medical records and interviews conducted with postpartum women during hospitalization in the rooming-in unit of the HU-USP. A second interview was conducted via telephone between the 21^st^ and 45^th^ day postpartum.

To verify the presence of probable PPD, the Edinburgh Postnatal Depression Scale (EPDS) was applied over the telephone because it is the most widely used, easy to apply, adapted and validated scale in our environment. Studies vary in the cut-off point used to characterize the presence of probable depression. We adopted a score greater than or equal to 10 because it is the most used.^14^

The PBQ was used to evaluate the mother-infant bond. This scale is considered a reliable instrument to detect dysfunctions in the mother-child relationship in the postpartum period. For this study, the 12 questions related to the bonding were applied. It is a Likert-type scale, with scores ranging from 0 to 5 for each question. The total score results from the sum of scores in each item; higher scores are related to worse bonds.^15,16^

The Stata 12 statistical software package was used for the analyses. After a descriptive analysis, the independent variables were categorized as presenting a higher or lower risk of altering the bonding. The nonparametric Mann-Whitney test was used to verify the potential association between the independent variables and the PBQ score and p values <0.05 were considered significant. Fischer’s test was used to verify the potential association between the independent variables and PPD, and the odds ratio (OR) was calculated by means of a binary univariate logistic regression analysis. The confidence interval was taken as 95% (CI95%) and p values <0.05 were significant. Correlation between the EPDS and PBQ score was tested using Spearman test.

The Ethics and Research Committee of HU-USP approved the study (Certificate of Appreciation for Ethics — CAAE 22464919.3.0000.0076).

## RESULTS

A total of 127 mother-infant dyads were studied. The mothers’ ages ranged from 18 to 43 years old and most lived with a partner (72.4%). Although the majority (70.9%) of the pregnancies were unplanned, most pregnant women discovered their pregnancy in the first trimester (86.6%) and had the minimum recommended number of prenatal visits (mean of eight visits). Only one pregnant woman in the sample had not had prenatal care. Half of the mothers (51.2%) had some complication during pregnancy, most often reporting urinary tract and/or gynecological infection. Only 3.3% of mothers reported a confirmed diagnosis of COVID-19 during pregnancy. EPDS score ranged from 0 to 26 points (mean=7.8). Prevalence of probable PPD (EPDS score ≥10) was 29.1% (Table 1).

**Table 1.** Maternal characteristics and pregnancy conditions.

Characteristics	n (%) or min-max (mean)
Age (years)	18–43 (27.5)
Marital status	
Single/separated	35 (27.6)
Married/stable union	92 (72.4)
Schooling	
Complete elementary	13 (10.2)
High/college	114 (89.8)
Planned pregnancy	
No	90 (70.9)
When she found out about the pregnancy	
1^st^ trimester	110 (86.6)
2^nd^/3^rd^ trimester	17 (13.4)
Number of prenatal visits	2–12 (8)
Complication(s) during pregnancy*	
Yes	65 (51.2)
Parity	
Primiparous	51 (40.2)
Previous abortion	
Yes	29 (22.8)
Feels supported by her partner	
Yes	108 (85.0)
Edinburgh scale score (EDPS)	0–26 (7.8)
Postpartum depression	
Absent (score <10)	89 (70.1)
Probable (score ≥10)	37 (29.1)
Unanswered	1 (0.8)

Data is expressed in number (%) or in minimum-maximum (mean). *Main complications reported: urinary/gynecological infection (25.7%), gestational diabetes (22%), hypertensive disease (21.3), syphilis/cytomegalovirus infection (4.7%), COVID-19 (3.3%), and others (8.9%). Many mothers reported more than one complication.

Regarding newborns, there was a predominance of females (55.1%), half were born by cesarean delivery, only 3.1% were premature, and only one was born with low weight. Most did not present perinatal complications (82.7%), went to a rooming-in unit during the first 24 hours of life (95.3%), and were discharged together with the mother (Table 2).

**Table 2.** Birth conditions and newborns’ characteristics.

Characteristics	n (%) or min-max (mean)
Type of delivery	
Normal/forceps	64 (50.4)
Cesarean section	63 (49.6)
Sex	
Female	70 (55.1)
Birth weight (grams)	2330–4300 (3299)
Gestational age	
Term	121 (95.3)
Preterm	4 (3.1)
No information	2 (1.6)
Breastfed in the delivery room	
Yes	46 (36.2)
Went to rooming-in in the first 24 hours of life	
Yes	121 (95.3)
Perinatal complication(s)*	
Yes	22 (17.3)
Discharge from the maternity ward together with the mother	
Yes	96 (75.6)
No	5 (3.9)
No information	26 (20.5)

Data is expressed in number (%) or in minimum-maximum (mean). *Main complications: neonatal jaundice (7.4%), respiratory distress (5.7%), hypoglycemia (3.3%), others (0.8%) PBQ: Postpartum Bonding Questionnaire.

**Figure 2.**
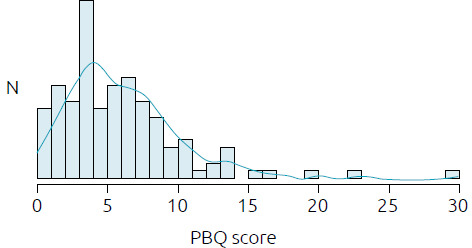
Distribution of the Postpartum Bonding Questionnaire scores.

The PBQ score presented a non-parametric distribution and ranged from 0 to 30, with a median of 6.0 (Figure 2). Analyzing the maternal characteristics and prenatal conditions (Table 3) and birth conditions (Table 4), we found that the variables that proved to be associated with the PBQ score and a higher risk of impaired bonding were: unplanned pregnancies (p=0.004) and the presence of probable PPD (p=0.001).

PBQ: Postpartum Bonding Questionnaire.

**Table 3.** Associations between maternal characteristics and pregnancy conditions with mother-infant bonding (Postpartum Bonding Questionnaire score) and postpartum depression.

	PBQ score					Postpartum depression			
	n (%)	Mean	Median	SD	p-value*	Absent	Probable	p-value^†^	OR (95%CI)
						89 (70.1%)	37 (29.1%)		
Maternal age (y)									
Under 20	12 (9.5)	5.67	5.50	2.01	0.536	8	4	0.746	1.22 (0.34–4.35)
20 or above	115 (90.5)	6.43	6.00	4.72		81	33		
Maternal schooling									
Elementary school	13 (10.2)	6.61	4.00	4.87	0.474	12	0	**0.018**	0.08 (0.0–1.43)
High school or college	114 (89.8)	6.33	6.00	4.52		77	37		
Marital status									
No partner	35 (27.6)	6.71	6.00	5.26	0.563	26	9	0.666	0.78 (0.32–1.88)
With partner	92 (72.4)	6.23	5.00	4.26		63	28		
Planned pregnancy									
Yes	37 (28.3)	4.70	4.00	3.12	**0.004**	27	9	0.665	1.35 (0.56–3.25)
No	90 (62.2)	7.04	6.00	4.86		62	28		
When she found out about the pregnancy									
1^st^ trimester	110 (86.6)	6.28	5.50	4.63	0.419	75	34	0.391	0.47 (0.13–1.75)
2^nd^/3^rd^ trimester	17 (13.4)	6.88	6.00	3.98		14	3		
Number of prenatal visits									
Insufficient (< 6)	8 (6.3)	6.50	7.50	3.74	0.930	1	0	1.000	0.79 (0.03–19.75)
Adequate (≥ 6)	116 (91.3)	6.36	5.00	4.57		88	37		
No information	3 (2.4)								
Complications during pregnancy									
Yes	65 (51.2)	6.09	5.00	4.61	0.408	48	16	0.330	0.65 (0.30–1.41)
No	62 (48.8)	6.65	6.00	4.49		41	21		
Parity									
Primiparous	51 (40.2)	6.78	6.00	5.24	0.742	33	18	0.239	1.61 (0.75–3.49)
Multiparous	76 (59.8)	6.08	5.50	4.02		56	19		
Previous abortion(s)									
Yes	29 (22.8)	5.59	4.00	3.74	0.336	21	7	0.644	0.75 (0.29–1.97)
No	98 (77.2)	6.59	6.00	4.75		68	30		
Support from partner									
Yes	108 (85.0)	6.15	5.00	4.40	0.176	78	29	0.273	1.96 (0.71–5.35)
No	19 (15.0)	7.58	7.00	5.26		11	8		
Postpartum depression									
Probable	37 (29.1)	8.73	7.00	5.96	**0.001**	–	–	–	–
Absent	89 (70.1)	5.40	4.00	3.41					

*Mann-Whitney test; ^†^Fischer’s test. PBQ: Postpartum Bonding Questionnaire; OR: odds ratio; SD: standard deviation; In bold: significant p value.

**Table 4.** Association between newborn characteristics and birth conditions with mother-infant bonding (Postpartum Bonding Questionnaire score) and postpartum depression.

	PBQ score (n=127)					Postpartum depression (n=126)			
	n (%)	Mean	Median	SD	p-value*	Absent	Probable	p-value^†^	OR [IC95%]
						89 (70.1%)	37 (29.1%)		
Sex									
Female	70 (55.1)	6.44	6.00	4.71	0.776	51	18	0.434	1.42 [0.66–3.06]
Male	57 (44.9)	6.26	5.00	4.37		38	19		
Type of delivery									
Normal/forceps	64 (50.4)	6.23	6.00	4.27	0.911	45	19	1.000	0.97 [0.45–2.09]
Cesarean section	63 (49.6)	6.49	5.00	4.84		44	18		
Gestational age									
Term	121 (95.3)	6.39	6.00	4.54	0.209	86	34	0.579	2.53 [0.34–18.69]
Preterm	4 (3.1)	4.00	2.50	4.24		2	2		
No information	2 (1.6)								
Breastfed in the delivery room									
Yes	46 (36.2)	5.87	5.50	4.07	0.474	36	10	0.223	1.83 [0.79–4.25]
No	81 (63.8)	6.64	6.00	4.78		53	27		
Rooming-in in the first 24 hours									
Yes	121 (95.3)	6.405	6.00	4.60	0.664	85	35	1.000	1.21 [0.21–6.93]
No	6 (4.7)	5.50	4.00	3.27		4	2		
Perinatal complications									
Yes	22 (17.3)	6.32	5.50	4.56	0.926	14	8	0.447	1.48 [0.56–3.89]
No	105 (82.7)	6.37	6.00	4.56		75	29		
Discharge with the mother									
Yes	96 (75.6)	6.52	6.00	4.53	0.524	64	31	1.000	1.37 [0.22–8.66]
No	5 (3.9)	8.00	7.00	5.24		3	2		
No information	26 (20.5)								

*Mann-Whitney test; ^†^Fischer’s test. PBQ: Postpartum Bonding Questionnaire; OR: odds ratio; SD: standard deviation. PBQ: Postpartum Bonding Questionnaire; EDPS: Edinburgh Scale score.

**Figure 3.**
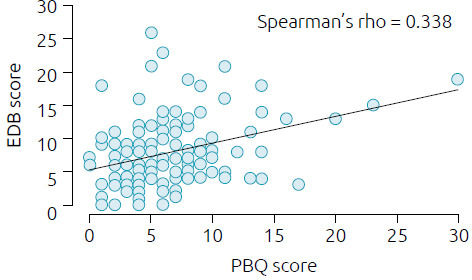
Correlation between the Postpartum Bonding Questionnaire score and the Edinburgh Scale score.

PBQ: Postpartum Bonding Questionnaire; EDPS: Edinburgh Scale score.

The EPDS score had a positive correlation with the PBQ score by Spearman’s test (rho=0.338, p<0.001; Figure 3). Only maternal schooling level was associated with probable PPD. However, this result is probably due to a bias related to sample composition and cannot be valued.

## DISCUSSION

We studied 127 postpartum women whose pregnancies occurred at a time of great global crisis, the first 18 months of the pandemic. High prevalence of probable PPD (29.1%) and unplanned pregnancies (70.9%) were found, which were associated with worse mother-infant bonding scores.

The pandemic brought a series of undesirable effects that, besides acute repercussions, might have long-term impacts. A considerable portion of children may evolve with impairment in their physical, socioemotional, and cognitive development, as a result of social isolation, school losses, and parental stress.^17^ There are several reasons for the increase in parental stress: fear and concerns related to COVID-19, socioeconomic losses, and changes in family routine, among others.^7,8^ In this sense, infants and postpartum women are a group that deserves special attention, since maternal mental health can increase perinatal risks and change DNA methylation. Consequently, it can alter the child’s response to stress, affect the organization of brain architecture and impair mother-infant bonding, representing a significant risk for the development of these children.^8,10,18^ Current studies seek to understand the impacts of the pandemic on child development. Shuffrey et al.,^19^ for example, reported that being born during the pandemic, regardless of whether the mother was infected or not, was associated with changes in motor and socioemotional development of infants at six months of age, indicating the importance of understanding the contextual factors in this situation. Therefore, assessing the bond between mother and baby plays an important role, since it is the basis of parenting and essential for the success of breastfeeding and for safe and responsive care, pillars of nurturing care considered by the World Health Organization as the basis for full child development.^4^ Changes in the establishment of the bond may have immediate repercussions, but also impact the development in the medium and long term.

There are few studies describing the effects of the pandemic on the bond, but it is inferred that factors involved in changes in the relationship that the mother establishes with the infant may include stress and psycho-emotional effects, in addition to the various changes imposed by the pandemic: less access to prenatal care, changes in protocols at birth, less contact between mother and newborn, and less possibility of family support for the puerperal woman resulting from social distancing. Some authors, comparing the pre and post-pandemic period, found worse scores on the scales that assess the bonding.^11,12^ Our study involved only women whose entire pregnancy and delivery were during the pandemic, allowing us to discuss which factors in the pandemic context would be related to the worsening of mother-infant bonding described in the literature.

We found that only maternal factors influenced the PBQ score. Higher scores, corresponding to worse bonds, were associated with the presence of probable PPD (p=0.001) and unplanned pregnancies (p=0.004). In our analysis, the scores on the Edinburgh scale were positively correlated with the PBQ score (Spearman’s rho=0.338; p<0.001), with the presence of PPD increasing the risk of impaired bonding. Several studies have already described PPD as a factor related to alterations in the mother-infant relationship, but what makes our result more worrying is that 29.1% of our sample had altered scores on the Edinburgh scale, showing a significant increase in this condition at the present time. A study conducted previously, involving 2,687 women and using the same screening instrument (EDPS) and the same cut-off score (risk of depression if score ≥10), reported a prevalence of 14% in our population.^20^ Therefore, our results indicate that the risk of PPD doubled during the pandemic, as reported by other authors.^9,10^ These findings warn of the need for a more cautious and attentive look at this issue by health professionals who care for *puerperae* and infants. The routine incorporation of screening scales for maternal depression in the care protocols should be considered by health services.

Several conditions are described that predispose to a higher risk of PPD: teenage pregnancy, low maternal education, low income, unplanned pregnancy, previous abortion, previous depression, exposure to violence, use of alcohol or illicit drugs, and lack of support or a stable partner.^21^ In our sample, only maternal schooling was related to a higher risk of PPD, but this result probably represents a bias and cannot be valued. Therefore, we assume that the high prevalence of PPD found was due to the context of insecurity secondary to the pandemic.

Puerperal depression deserves to be highlighted, both due to the increase in its prevalence and due to being a potentially modifiable factor through the health care offered. Prenatal care and delivery care routines can reduce its occurrence and, when not avoided, its early identification and approach can minimize the undesirable impacts. By interfering with the establishment of the bond, as verified in our study, puerperal depression can affect the child’s development. In a systematic review, Martucci et al.^22^ reported that maternal depression altered the exercise of parenting and was consequently correlated with high levels of emotional dysregulation and irritability in children, as well as delays in neuropsychomotor development. Another nationally based study of over 90,000 mothers found the same negative results on child development due to reduced physical and verbal interactions between mothers and infants, drawing attention to the need to place these children in contact with other adults who can stimulate them adequately.^23^ Considering the social distancing imposed by the pandemic, this protective effect of co-parenting on child development was possibly less frequent in children born in this period, aggravating the situation.

Other authors have already described the relationship between parental stress and early impairment to children. Provenzi et al.^24^ reported lower self-regulatory skills in 3-month-old infants during the pandemic due to maternal stress and anxiety. Despite the importance of caregivers’ emotional status, other aspects should be discussed.

Pregnancy planning was another factor that we found with an unfavorable outcome in the bond. This condition, like PPD, has also been more frequent in the current context. The risks, economic impacts, and uncertainties experienced during the pandemic have led women to choose to postpone pregnancies.^25^ However, reduced access to contraceptive methods was associated with an increase in unplanned pregnancies. The United Nations Sexual and Reproductive Health Agency^26^ estimates that, in the first year of the pandemic, there were approximately 1.4 million unplanned pregnancies, further contributing to the negative impacts on women’s mental health and perhaps to the increase in PPD cases in different countries.

Our results reinforce the importance of paying attention to women’s reproductive health care, prenatal care, and measures that promote maternal well-being and bonding, such as breastfeeding in the delivery room, skin-to-skin contact, and rooming-in. In this sense, it is important to point out that, although these practices are valued at the HU-USP, the pandemic changed the care protocols and only 36.2% of our mothers breastfed in the delivery room. It is also especially important that health professionals, including pediatricians, identify early signs of postpartum depression and refer *puerperae* to specialized care in order to ensure nurturing care and fully promote the child’s potential development.

Our study has some limitations. This is a cross-sectional study of a convenience sample. Therefore, it is not possible to infer causal relations. In addition, as the population studied was homogeneous from the sociodemographic point of view and data collection was performed online with self-reported instruments, it is possible that selection and information biases have occurred. Despite this, the paper reports important results and implications of the pandemic.

Among many other negative impacts, mental and emotional health impairments during the pregnancy-puerperium cycle will possibly have direct and indirect medium and long-term impacts on child development and should guide research in this field. The evaluation of the caregivers’ psycho-emotional conditions and the impact on children should be incorporated in best care practices. A longitudinal follow-up of children born during the pandemic for the early detection of problems and the adoption of interventions to support parenting are essential to mitigate the undesirable consequences.

## Data Availability

The database that originated the article is available with the corresponding author.
